# Abstracts

**Published:** 1980

**Authors:** 


### Abstracts

### South West Surgical Club Meeting, 25th and 26th April, 1980

Saunton Sands Hotel, Barnstable, and
Postgraduate Centre, North Devon District Hospital

### IS BONE SCANNING OF VALUE IN PATIENTS WITH BREAST CANCER

**G. R. Spencer, M. Khan, R. Seymour, C. D. Collins**
**Musgrove Park Hospital, Taunton**

150 patients are reviewed who had a bone scan carried out using technetium 99 methylene diphosphonate at the time of initial treatment for breast cancer. Of 101 patients with stage I/II breast cancer, only 2 (2%) had positive scans. In neither case was the management altered by the result of the scan. Five of the 99 patients who had negative bone scans died within 19 months. Because of the high cost of each test, the low yield of positive scans and high yield of false negatives, there seems to be no justification for routine staging bone scans using technetium in patients with stage I/II disease. It is proposed that routine osetrogen receptor assays would be of more value in determining prognosis and subsequent management.

In follow-up patients with skeletal symptoms, it was found that in 15/17 cases (90%), the diagnosis of a skeletal secondary could be made with radiology alone without resorting to a bone scan.

### WIDE LOCAL EXCISION IN BREAST CARCINOMA: A REVIEW OF THE RESULTS

**J. P. Partridge**
**North Devon District Hospital**

Wide local excision for carcinoma of the breast was done in 146 patients over a 12 year period. The five year survival was 71%, similar to the results of many series of other methods of treatment. 72 patients had post operative radiotherapy, 24 dying of the disease, whereas 74 had no radiotherapy, 10 dying of carcinoma of the breast and 11 of other causes. As many of this latter group had surgery within the past five years these figures are of no value. Local recurrence developed under the scar in the breast and/or glands in 30 patients (about 20%): 17 are alive and free of disease, and 13 died (3 of other causes). Two patients had carcinoma in both breasts, one alive and free of disease 10 and 7 years after the operations, the other 5½ and 3 years, both after wide local excision. In these women keeping their breast was doubly important. In only one patient was there a further carcinoma in the same breast of different histology. It seems therefore that, as 25% of women with early carcinoma of the breast already have bone metastases, and where evaluation of local node involvement clinically or by random sampling may give up to 30% error, wide local excision offers a reasonable way of treating early carcinoma of the breast, especially as fear of multicentric origin has proved unfounded.

### HEPATO-BILLARY IMAGING WITH HIDA

**G. R. Spencer, G. T. Watkin, C. Bird, A. M. Flynn, T. R. Brown, C. D. Collins**
**Musgrove Park Hospital, Taunton**

Technetium$^{99m}$ labelled diethyl-iminodiacetic acid (Tc$^{99m}$—HIDA) is a radiopharmaceutical which, when injected intravenously, is normally rapidly excreted into the bile, allowing the liver, gall-bladder and bile duct to be imaged with a gamma camera. HIDA imaging, which does not need special skill to carry out, has been successfully used to diagnose acute cholecystitis and to differentiate medical and surgical jaundice. Although it does not give as much anatomical detail as percutaneous transhepatic cholangiography and endoscopic retrograde cholangio-pancreatography, it is a safe, non-invasive test, free from the serious complications of cholangitis, pancreatitis, bleeding and biliary peritonitis which may follow the other procedures. In order to assess the value of HIDA imaging 20 jaundiced patients with bilirubin levels of 26-330 in moles/L and whose ultimate diagnosis became known were reviewed. HIDA imaging correctly diagnosed 15/20 (75%) patients. In 4 patients the imaging was equivocal and in 1 patient with primary biliary cirrhosis it was definately misleading. It is concluded that, providing the surgeon carries out a pre-operative cholangiogram routinely to provide anatomical localisation of the lesion, much time could be saved pre-operatively by using a HIDA imaging rather than waiting for more complicated and invasive investigations.

### PAROTID TUMOURS — DIAGNOSIS AND TREATMENT

R. G. Hughes and T. J. Lyons
Bristol Royal Infirmary

Between 1968 and 1978 ninety eight salivary gland tumours were treated surgically in the United Bristol Hospitals. The commonest tumour was pleomorphic adenoma occurring in the parotid gland, accounting for 56 (58%) of the cases. These lesions were treated by superficial parotidectomy (40) or local enucleation without radiotherapy (16). The recurrence rates for the two operations were 5% and 25% respectively, all recurrences occurring at least five years after the initial operation. While superficial parotidectomy had a low recurrence rate it had a high incidence of minor neurological sequelae. Gustatory sweating occurred in over 30% of the patients after parotidectomy and in none after enucleation. Only 30% of parotidectomy patients declared themselves normal compared to 60% following enucleation. Pre-operative diagnosis was on clinical findings in all cases and, additionally, needle aspiration biopsy in 34. Aspiration biopsy was 95% accurate but the two recurrences following parotidectomy had undergone this test.

Aspiration biopsy is useful in identifying the pathology accurately prior to operation but may predispose to recurrence later. Superficial parotidectomy is the treatment of choice for pleomorphic adenomas of the parotid gland. Although there is a high incidence of neurological sequelae these are usually minor and of little consequence to the patients.

### DRILL BIOPSY OF BREAST CARCINOMA PRE-OPERATIVE HISTOLOGICAL DIAGNOSIS

John R. Barker
North Devon District Hospital

Most surgeons are unwilling to proceed to mastectomy without an histological diagnosis. Traditional frozen section and excision biopsy have many drawbacks and a pre-operative diagnosis would be ideal. Fine needle aspiration requires an experienced cytologist and Tru-cut, in the author's hands, often results in an inadequate specimen. A high speed air drill produces good specimens from which the pathologist has no difficulty in producing a diagnosis. Between January 1974 and January 1980 this has been used by the author. All suspicious lumps were drilled, cysts having been excluded by fine needle aspiration. All patients with a positive biopsy for carcinoma had a mastectomy; those with a negative result, but a residual lump, had an excision biopsy as a day case. A total of 210 biopsies were performed. No major haematoma nor infection resulted from the biopsy. 115 were reported as malignant and these were all confirmed at mastectomy. 95 were reported as benign and 5 were found to be malignant at excision biopsy. There were no false positives and an overall accuracy of 97.6%.

### MORTALITY OF OPEN PROSTATECTOMY: A PERSONAL SERIES

D. I. Stirk
North Devon District Hospital

In a personal series of 691 patients, 223 of whom were graded as Group 3 under the Blandy classification, the overall mortality of open prostatectomy was 2.5%. 479 patients were aged 50—75 years and in them the mortality was 0.6%. The mortality was 6.6% in the remainder whose ages were 76 to over 90 years.

The results are favourable compared with other open prostatectomy series and compare well with the results of series of transurethral resections. The general conclusion is that the method of prostatectomy is less important than the pre- and post-operative care and the prevention of infection. A short, active pre-operative assessment and preparation does much to eliminate post-operative complications. In the avoidance of infection it is imperative that the closed system catheter drainage is not disconnected. A technique using post-operative diuretics can avoid the need for washouts and is a vital part of the post-operative care. It is suggested that epidural spinal anaesthesia producing a moderate degree of hypotension and using the 'topping-up' procedure post-operatively to relieve pain contributes greatly to improved results.

As can be seen from the mortality figures, age plays a most important part in the mortality of prostatectomy, and patients aged over 80 years are particularly at risk. Of the 17 deaths in this series, 9 were due to coronary insufficiency and 5 due to respiratory infection; in only one of the former was there a history of cardiac ischaemia.

### FEMORO-POPLITEAL BYPASS: A TRIAL OF VEIN VERSUS GORE-TEX

John R. Barker
North Devon District Hospital

Gore-tex, polytetrafluoroethylene, was introduced into the United Kingdom as a vascular replacement in 1975. Experience had suggested good long term patency in femoro-popliteal bypass, when the long saphenous vein was unavailable or inadequate. Between November 1977 and May 1979 a study was done on 47 grafts in 45 patients with rest pain and/or incipient gangrene and femoral artery block. A Gore-tex graft was used in 24 when the long saphenous vein was absent or inadequate; the other 23 had a reverse saphenous graft implanted. A below knee anastomosis was used in all the patients. The patients were equally matched as regards age, sex, diabetes, smoking habits, and numbers requiring minor amputation at the time of the graft.

There were no operative deaths, but 7 died during the follow-up period, 2 who had a vein and 5 a Gore-tex graft. The maximum follow-up was 18 months with a minimum of 6 months. 2 veins failed, resulting in 1 major amputation, and 9 Gore-tex grafts failed resulting in 6 major amputations. At 1 month, 2 out of 23 vein grafts had failed whereas at this stage only 1 of 24 Gore-tex had failed. At 18 months 2 out of 15 vein grafts had failed, giving an 87% patency rate, whereas 5 out of 11 Gore-tex grafts had failed, giving a patency rate of 56%.

### OESOPHAGEAL BRUSH CYTOLOGY IN THE DIAGNOSIS OF CARCINOMA OF THE OESOPHAGUS

Neil Mortensen and Elizabeth Mackenzie
Departments of Surgery & Cytology
Southmead Hospital, Bristol

Brush cytology (BC) may increase the accuracy of diagnosing oesophageal carcinoma. We have studied the value of BC in the diagnosis of 101 strictures of the oesophagus over a 15 month period using a cytology service with experience on gynaecological but not oesophageal cytology. BC specimens were read 'blind' and this diagnosis compared with that of radiology, endoscopy, and biopsy. BC gave a correct diagnosis in 86% of 36 malignant strictures, comparing favourably with biopsy (80%), endoscopy (83%) and radiology (80%).

When combined with biopsy accuracy rose to 92% (Table). Flexible or rigid endoscopy were equally effective for adequate sampling. Of 64 benign strictures examined by BC there were 3 false positives (4.6%), 3 described as suspicious and 6 technical failures (82% correct). In BC slide survey (10 benign, 10 malignant) sent to eight hospitals in the region, average false negative diagnosis was 12%, and false positive 3.6%. Four of the centres had little previous experience and only two extensive experience of oesophageal cytology. We conclude that BC is a useful and simple technique in the investigation of oesophageal strictures. There is a wide variation in the use of BC in our region, but it is within the capability of most pathology departments.

**TABLE**

**Results of cytology in 36 malignant strictures**

| | *Total* | *Correct* | *False Negative* | *Suspicious* | *Fail* |
|---|---|---|---|---|---|
| Cytology | 36 | 31 (86%) | 2 (5.5%) | 2 (5.5%) | 1 (3%) |
| Biopsy | 26 | 21 (81%) | 2 (8%) | 2 (8%) | 1 (3%) |
| Cytol. + Biopsy | 25 | 23 (92%) | 2 (4%) | 1 (4%) | 0 (0%) |

### THE INVESTIGATION OF SUSPECTED PANCREATIC MALIGNANCY: AN AMERICAN EXPERIENCE

**M. J. Cooper and R. G. Hughes**

**University Department of Surgery**
**Southmead Hospital, Bristol**

Carcinoma of the pancreas is increasing in incidence in the USA and Western Europe. At present the only hope for cure is radical surgery, which all too often is precluded by the failure of early diagnosis. To examine the value of the various diagnostic techniques available, a prospective 'diagnostic protocol' was started in Chicago. Over a four-year period 186 patients have been entered in the protocol, the criteria for entry being obstructive jaundice, unexplained weight loss ($>$10% body weight), upper abdominal or back pain, and any patient with a suspected pancreatic mass. Investigations included ultrasound scan, radioisotope scan, computer tomography (CT), duodenal drainage studies, duodenal cytology, ERCP, and arteriography. The results were compared with laparotomy findings (146 patients), or with follow-up (minimum one year). Seventy-three patients (39%) were found to have pancreatic cancer, 31 (17%) other cancers, 24 (13%) chronic pancreatitis, and 58 (31%) other benign diseases. The diagnostic accuracy of the tests was: ultrasound 83%, isotope scan 66%, CT 79%, duodenal drainage 65%, cytology 98%, ERCP 98%, and arteriography 73%. The results show that an ultrasound scan is the best initial test in a patient with suspected pancreatic cancer, and that ERCP, combined with cytology, is most likely to provide a definitive diagnosis.

